# Molecular evolution of octopamine receptors in *Drosophila*

**DOI:** 10.1093/g3journal/jkaf289

**Published:** 2025-12-06

**Authors:** Mengye Yang, Jolie A Carlisle, Ben R Hopkins, Mariana F Wolfner

**Affiliations:** Department of Molecular Biology and Genetics, Cornell University, Ithaca, NY 14853, United States; Department of Molecular Biology and Genetics, Cornell University, Ithaca, NY 14853, United States; Department of Evolution and Ecology, University of California, Davis, CA 95616, United States; Department of Molecular Genetics and Microbiology, University of Florida, Gainesville, FL 32610, United States; Department of Molecular Biology and Genetics, Cornell University, Ithaca, NY 14853, United States

**Keywords:** *Drosophila*, octopamine receptor, molecular evolution, positive selection, conservation, reproduction

## Abstract

Octopamine (OA), the insect analog of noradrenaline, plays important roles in diverse behavioral and physiological processes, from modulating fight-or-flight behavior to regulating postmating ovulation. In *Drosophila*, 6 OA receptors have been identified: Oamb, Octα2R, Octβ1R, Octβ2R, Octβ3R, and Oct-TyrR, and they have been linked to different behavioral and physiological processes. Here, we investigated the evolutionary characteristics of these receptors across *Drosophila* species. We found that OA receptors are generally found as single-copy genes. Notably, Octβ2R and Octβ3R exhibit positive selection within the *melanogaster* species group, though in different structural regions from one another. The positively selected sites in Octβ2R are exclusively located in regions important for ligand binding, whereas those in Octβ3R are predominantly found in regions crucial for signal transduction. Interestingly, Octβ2R remains highly conserved outside the *melanogaster* species group, so the detection of positive selection in its ligand binding-related domains within this clade raises the possibility that it has evolved an additional, *melanogaster*-specific ligand interaction, among other potential reasons. These findings highlight the evolutionary flexibility of aminergic signaling and suggest lineage-specific adaptations of OA receptor function in *Drosophila*, likely shaped by lineage-specific selective pressures.

## Teaser

Octopamine is a chemical messenger in insects, analogous to noradrenaline in vertebrates, and influences many processes such as fight-or-flight responses, locomotion, and reproduction. In fruit flies and other insects, multiple octopamine receptors detect and respond to this signal. Yang *et al*. explored how these receptors have changed over millions of years across various fly species. The authors found that while some receptors remain similar, others have evolved more rapidly, possibly due to changes in the insects’ lifestyles, environments, or other biological demands. Understanding these differences can help reveal how insects fine-tune their behaviors and adapt to diverse challenges.

## Introduction

Biogenic amines are small signaling molecules that play multiple roles in regulating physiological and behavioral functions across both mammals and insects. In mammals, noradrenaline and adrenaline mediate fight-or-flight responses and other key processes. In insects, however, these catecholamines are absent or present only at very low levels. Instead, insects rely on structurally and functionally analogous molecules: tyramine (TA) and octopamine (OA). The amino acid tyrosine is first converted to TA by tyrosine decarboxylase, and TA is subsequently converted to OA by TA β-hydroxylase. Although TA serves as the biosynthetic precursor to OA, both can act as independent neurotransmitters (Roeder 2005). In *Drosophila melanogaster* (fruit fly), OA function has been particularly well studied, revealing its pleiotropic roles in diverse processes including locomotion, aggression, learning, memory, sleep, energy intake and expenditure, and reproduction (Roeder 2005, 2020; Farooqui 2012; White et al. 2021).

OA exerts its diverse physiological and behavioral effects by binding to and activating its receptors, all of which belong to the G protein-coupled receptor (GPCR) family. In *Drosophila*, these receptors are classified into 4 groups based on their structural and signaling similarities to vertebrate adrenergic receptors: Oamb (α-adrenergic-like OA receptor), Octα2R (α_2_-adrenergic-like OA receptor), 3 β-adrenergic-like receptors (Octβ1R, Octβ2R, and Octβ3R), and the OA/TA receptor Oct-TyrR (Evans and Maqueira 2005; Qi et al. 2017). Despite being functionally related proteins belonging to the same protein family, the relationships among the OA receptors remain unresolved. Previously, 2 independent evolutionary studies constructed phylogenetic trees of biogenic amine receptors across species. As expected, β-adrenergic-like OA receptors clustered together; however, the phylogenetic and evolutionary relationships with and among the other OA receptors remained unclear due to weak branch support (Qi et al. 2017; Zhang et al. 2023). Upon activation, Oamb promotes increases in intracellular calcium and cyclic-AMP (cAMP) levels and has been implicated in processes such as follicle rupture, ovulation, sperm storage, sleep/wake regulation, appetitive learning and memory, insulin-like peptide transcription, and male aggression (Han et al. 1998; Balfanz et al. 2005; Lee et al. 2009; Crocker et al. 2010; Avila et al. 2012; Burke et al. 2012; Kim et al. 2013; Luo et al. 2014; Deady and Sun 2015; Huetteroth et al. 2015). Octα2R, by contrast, reduces cAMP production and has been linked to locomotion, grooming behavior, and starvation-induced hyperactivity (Qi et al. 2017; Nakagawa et al. 2022). The OctβRs predominantly signal through cAMP (Sabandal et al. 2020). Octβ1R is involved in olfactory learning, exercise adaptation, and hunger-driven modulation of female receptivity (Sabandal et al. 2020; Sujkowski et al. 2020; Sun et al. 2023). Octβ2R regulates ovulation, locomotor activity, anesthesia-resistant memory, and sleep, as well as stimulates synaptic growth, an effect antagonized by Octβ1R (Koon et al. 2011; Koon and Budnik 2012; Wu et al. 2013; Lim et al. 2014; Li et al. 2015; Zhao et al. 2021). Octβ3R has been implicated in metamorphosis and appetitive motivation (Zhang et al. 2013; Ohhara et al. 2015). Finally, Oct-TyrR is sensitive to both TA and OA, with TA being slightly more potent in inhibiting adenylate cyclase activity, while OA more strongly stimulates calcium signaling (Robb et al. 1994). Oct-TyrR has been shown to affect chemotaxis behavior and startle responses by modulating downstream dopaminergic neuron activity (Ma et al. 2016).

Genes associated with reproduction have often been observed to evolve more rapidly than nonreproductive genes, sometimes exhibiting elevated sequence divergence potentially driven by sexual selection, sexual conflict, or relaxed selective constraints (Swanson and Vacquier 2002; Clark et al. 2006; Wilburn and Swanson 2016; Dapper and Wade 2020; Carlisle and Swanson 2021; Patlar et al. 2021). For example, in *Drosophila melanogaster*, male seminal fluid proteins trigger the female postmating response, a potential battleground of male × female interaction that can drive elevated sequence evolution of seminal fluid proteins. Indeed, 7% to 12% of male seminal fluid proteins show signatures of positive selection potentially driven by sexual selection or sexual conflict, although their rapid diversification can also be driven by relaxed selection (Swanson et al. 2001a; Haerty et al. 2007; Wong and Wolfner 2012; Patlar et al. 2021). Although fewer studies have addressed the evolution of female reproductive proteins, growing evidence suggests that positive selection also acts on the female side (Swanson et al. 2001b; Galindo et al. 2003; McGeary and Findlay 2020; Moyle et al. 2021). Given that multiple OA receptors are involved in reproductive processes (Lee et al. 2009; Avila et al. 2012; Lim et al. 2014; Deady and Sun 2015; Li et al. 2015; Sun et al. 2023), it is of particular interest to explore the evolutionary dynamics of this receptor group.

Here, we identified OA receptor orthologs across 27 *Drosophila* species and detected 2 lineage-specific tandem duplication events using synteny analysis. Nevertheless, OA receptors are generally maintained as single-copy genes. Using a suite of molecular evolution analyses, we found evidence of positive selection on Octβ2R and Octβ3R within the *melanogaster* species group (in this manuscript specifically referring to members of the *melanogaster*, *takahashii*, *suzukii*, *elegans*, and *rhopaloa* subgroups). Interestingly, we did not detect evidence for positive selection of Octβ2R in the *virilis*-*repleta* radiation nor in mosquitoes, suggesting that the positive selection observed in residues important for ligand binding within *melanogaster* species may reflect lineage-specific selective pressures. These selective pressures could include sexual selection on reproductive function, pathogen-mediated selection, ecological adaptation involving receptor modulation, or perhaps co-option to bind a ligand other than OA. Taken together, these results reveal distinct evolutionary trajectories shaping OA receptor evolution.

## Materials and methods

### Synteny analysis and tandem duplication detection

To identify potential tandem duplications of OA receptors, we first determined their syntenic regions by focusing on the 3 closest upstream and downstream genes flanking each OA receptor in the *D. melanogaster* genome, as identified using the JBrowse map within FlyBase (flybase.org) (Öztürk-Çolak et al. 2024). Using 3 neighboring genes provides sufficient local context to detect tandem duplications without extending into unrelated genomic regions. We then identified orthologs of these 7 genes (including the focal OA receptor) in 27 *Drosophila* species whose genomes had been annotated by the automated NCBI Gnomon prediction pipeline. The *D. melanogaster* sequence of each gene was used as the query in the tBLASTn search against the annotated transcriptomes for each species. The species we tested were: *melanogaster*, *simulans*, *mauritiana*, *sechellia*, *erecta*, *yakuba*, *santomea*, *teissieri*, *takahashii*, *suzukii*, *subpulchrella*, *biarmipes*, *elegans*, *rhopaloa*, *ficusphila*, *kikkawai*, *ananassae*, *persimilis*, *pseudoobscura*, *willistoni*, *busckii*, *grimshawi*, *mojavensis*, *hydei*, *virilis*, *innubila*, and *nasuta*. We also performed reciprocal tBLASTn searches against *D. melanogaster* transcriptomes to strengthen the confidence in ortholog prediction. Their chromosomal locations were confirmed using the NCBI Genome Data Viewer, and the OA receptor copy number was determined for each species. For species with more than 1 copy of an OA receptor, RNA sequencing (RNA-seq) exon coverage tracks were inspected on the species' genome browsers to confirm transcription of each identified paralog. For OA receptors, at least 1 side of the flanking genes showed some level of conservation, supporting their placement within the expected genomic context. For flanking genes that differed from those in *D. melanogaster*, we used Gnomon predictions available on NCBI to confirm their identities. Synteny metrics are provided in Supplementary File 1, and more details are available in Supplementary File 6.

High-throughput methods such as those used in OrthoDB (Tegenfeldt et al. 2025) and DrosOMA (Thiébaut et al. 2024) provide large-scale resources for ortholog annotation, but they rely on automated pipelines that can misclassify relationships, particularly in cases of gene duplication or lineage-specific loss (Carlisle et al. 2024). Our approach involves nonautomated, detailed, and targeted characterization of receptor genes across species. While our results largely agree with OrthoDB and DrosOMA for most OA receptors existing as single-copy genes in species examined, these databases did not reliably find the duplicates that we detected. The duplicate in *D. busckii* of *Octβ3R* was not detected in either DrosOMA or OrthoDB, and the duplicate in *D. sechellia* of *Octα2R* was not detected in DrosOMA but was detected in OrthoDB. This inconsistency between our results and ortholog predictors and the inconsistency between ortholog predictors themselves highlight the value of our detailed, targeted investigations, such as those presented here.

### Molecular evolution analyses

We performed Phylogenetic Analysis by Maximum Likelihood (PAML) analyses on each OA receptor across 15 species within the *melanogaster* species group, as listed in Supplementary Fig. 4. Only isoforms detectable in more than 10 species by Gnomon prediction were included in the analysis. For Octα2R, *D. sechellia* (*Dsec*) orthologs were excluded due to the presence of 2 copies in this species. Protein sequence alignments were generated by Clustal Omega (Madeira et al. 2024), which were then converted into codon-based DNA alignments with PAL2NAL (Suyama et al. 2006). Protein alignment statistics can be found in Supplementary File 7. Maximum likelihood trees were constructed with reference to 2 established phylogenies: a high-confidence phylogeny of 155 *Drosophila* species (Suvorov et al. 2022) and an additional tree including *D. santomea* (Hopkins et al. 2024) to determine its phylogenetic position. The codeml program from the PAML package (Yang 2007) was used to calculate an overall *ω* estimate for the whole sequence under model M0 and to perform site tests by comparing model M8, which allows for a class of sites with *ω* > 1, against the null models M7 and M8a using likelihood ratio tests (Yang et al. 2000; Swanson et al. 2003). The “cleandata” option was enabled in codeml to remove alignment sites with gaps or ambiguous data. For genes where model M8 provided a significantly better fit than models M7 and M8a, the Bayes empirical Bayes (BEB) approach was applied to identify positively selected sites (PSSs) at a 0.9 confidence level.

For Octβ2R, PAML analyses were also performed within the *D. virilis*-*repleta* radiation and across mosquito species, following the similar procedures described above. Ortholog prediction was conducted in the same manner as for the 27 *Drosophila* species (see the “Synteny analysis and tandem duplication detection” section), and PAML analyses were carried out as described in the preceding paragraph. Within the *virilis*-*repleta* radiation, 7 species (*virilis*, *novamexicana*, *hydei*, *navojoa*, *mojavensis*, *arizonae*, and *montana*) were chosen based on the availability of transcriptome data. In mosquitoes, PAML tests were performed separately within the *Cellia*, *Anophelinae*, and *Culicidae* lineages. The species included in these analyses, along with their phylogenetic relationships (Neafsey et al. 2015; Suvorov et al. 2022), are shown in Fig. 4b.

Mixed Effects Model of Evolution (MEME) analyses were conducted using the Datamonkey Adaptive Evolution Server (Weaver et al. 2018) with default parameters and 100 resamples. The maximum likelihood trees and DNA alignments used in the PAML analyses were also used as input for these analyses.

### Codon usage bias and GC content analysis

Coding DNA sequences (CDS) of Octβ2R and Octβ3R from 15 species in the *D. melanogaster* species group were analyzed to assess codon usage bias and GC content. Sequences were first cleaned by removing gaps (-). Codon usage metrics were calculated using the coRdon R package (v1.20.0), including the effective number of codons (ENC) to quantify overall codon usage bias. Overall guanine+cytosine (GC) content and GC content in the third codon position (GC3) were computed using the seqinr R package (v4.2.36). For GC3, the third nucleotide of each codon was extracted and the proportion of G or C nucleotides was calculated per sequence.

ENC values range from 20 (extreme codon bias) to 61 (no bias), with intermediate values indicating moderate codon usage bias. GC and GC3 metrics were used to evaluate potential nucleotide composition shifts that might influence *ω* estimates. All analyses were performed in R (v4.3.2) on gap-free, full-length CDS alignments.

## Results and discussion

### OA receptors are found as single-copy genes in most of the *Drosophila* genomes we analyzed

**Fig. 1.**
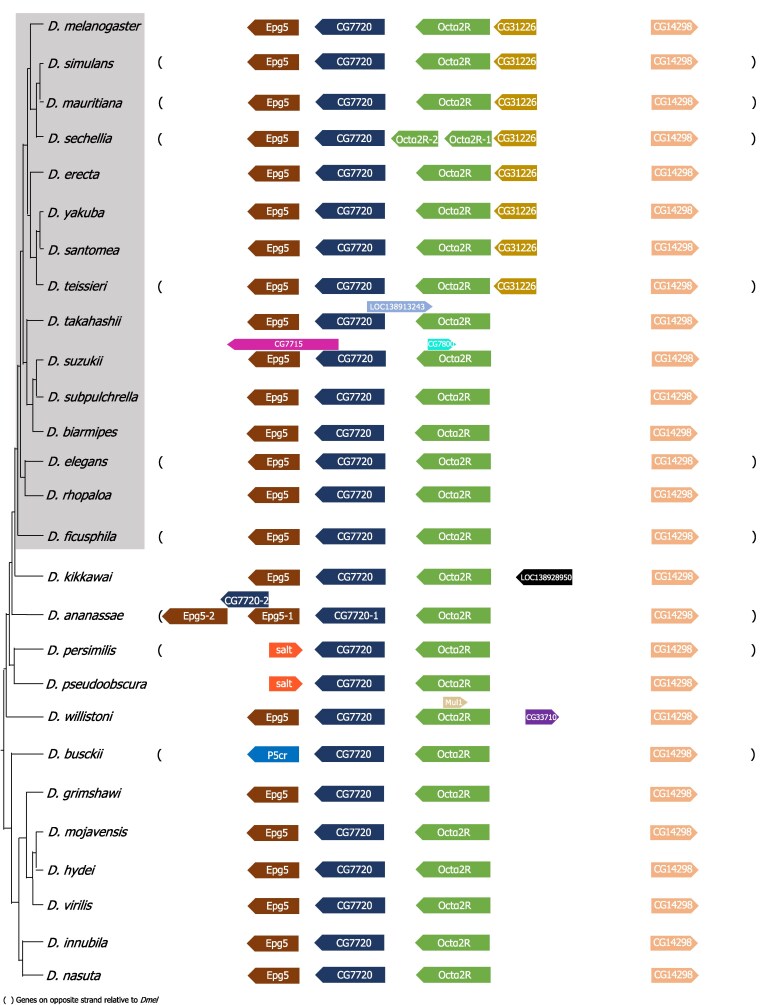
Syntenic region of *Octα2R* across *Drosophila* species. The phylogeny is based on Suvorov et al. (2022) and Hopkins et al. (2024). Surrounding gene names correspond to orthologs in *D. melanogaster*. The species within the gray box represent members of the *melanogaster* species group, with membership determined by their phylogenetic relatedness as described by Suvorov et al. (2022).

**Fig. 2.**
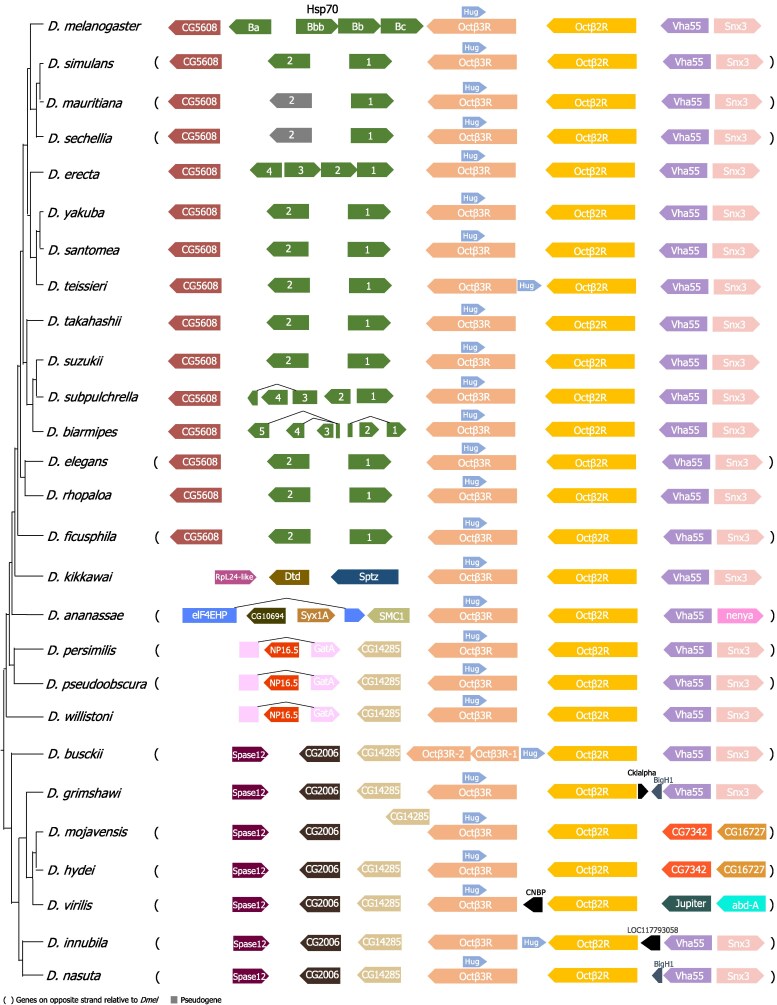
Syntenic region of *Octβ2R* and *Octβ3R* across *Drosophila* species. The phylogeny is based on Suvorov et al. (2022) and Hopkins et al. (2024). Surrounding gene names correspond to orthologs in *D. melanogaster*.

To examine the evolutionary history of OA receptors, we first identified the orthologs of OA receptors across 27 *Drosophila* species with available transcriptome data, which, combined with the NCBI Gnomon gene predictions, provides comprehensive and well-supported receptor annotations. Searches against transcriptomes, rather than genomes, are more sensitive to detect paralogs since expressed coding sequences do not have introns that complicate genome-based searches and the database that is being searched against is much smaller. By examining syntenic regions, we found that most OA receptor family members are retained as single-copy genes across species (Figs. 1 and 2, Supplementary Figs. 1 to 3, Supplementary File 1), reflecting evolutionary conservation and functional constraint. This is likely important for maintaining a tightly regulated neuromodulatory system, where altered gene dosage or expression levels could lead to signaling imbalances and physiological dysfunction.

Interestingly and in contrast, the *D. sechellia (Dsec)* genome possesses 2 copies of *Octα2R* (Fig. 1), and the *D. busckii* (*Dbus*) genome has 2 copies of *Octβ3R* (Fig. 2). Although the evolutionary forces underlying this variation in the OA receptor copy number are unknown, it is intriguing that both *D. sechellia* and *D. busckii* have adapted to thrive on toxic hosts: *D. sechellia* feeds on *Morinda* fruit (Louis and David 1986; Legal et al. 1992), while *D. busckii* consumes rotting vegetables such as potatoes (Atkinson 1977; Būda et al. 2009). This raises the possibility that OA signaling may play a role in adaptation to environmental conditions through lineage-specific diversification or amplification. Since dopamine and OA signaling interact functionally in flies (Schwaerzel et al. 2003; Burke et al. 2012; Ma et al. 2016; Sabandal et al. 2020), and the dopaminergic system is crucial for *D. sechellia*'s reproductive success and specialization on its toxic host (Lavista-Llanos et al. 2014), it is plausible that OA signaling may also have contributed to such species-specific adaptations. Gene copy number changes have been linked to ecological adaptation in other species. For example, human populations with high-starch diets exhibit an increased amylase gene copy number relative to those with lower-starch diets (Perry et al. 2007).Together, these findings raise the possibility that OA receptor copy number variation may contribute to ecological specialization and environmental adaptation. Alternatively, this variation may reflect reduced selective restraint on these genes in these species, leading to toleration of duplication.

### Octβ2R and Octβ3R have undergone positive selection in the *D. melanogaster* group

We then used PAML to investigate whether any of the OA receptors contain PSSs (Yang 2007), focusing our analysis on species within the *melanogaster* group (Fig. 1, Supplementary Fig. 4), which corresponds to Clade 4 in the phylogeny of Suvorov et al. (2022). Because the functions of these genes have been characterized in *D. melanogaster*, limiting our selection analyses to this clade allows for evolutionary insights that are more directly relevant to the experimentally studied functions. Model M0 was used to estimate the overall *d*_N_*/d*_S_ (*ω*) ratio across the entire protein-coding sequence of each gene; and M7 vs. M8 and M8a vs. M8 comparisons (where M7 and M8a are neutral models and M8 is a model that allows for PSSs) were used to identify genes evolving under positive selection and identify specific PSSs (Yang et al. 2000; Swanson et al. 2003). The M8a null model explicitly includes neutrally evolving sites, and the M8a/M8 comparison is therefore a more sensitive alternative to M7/M8 and less likely to yield false positives. We consider a gene to be under positive selection only if both comparisons are significant. For genes under positive selection, we use the posterior probabilities from the BEB test included in the output for codeml's M8 for identifying sites under positive selection with a posterior probability threshold of 0.9.

**Fig. 3.**
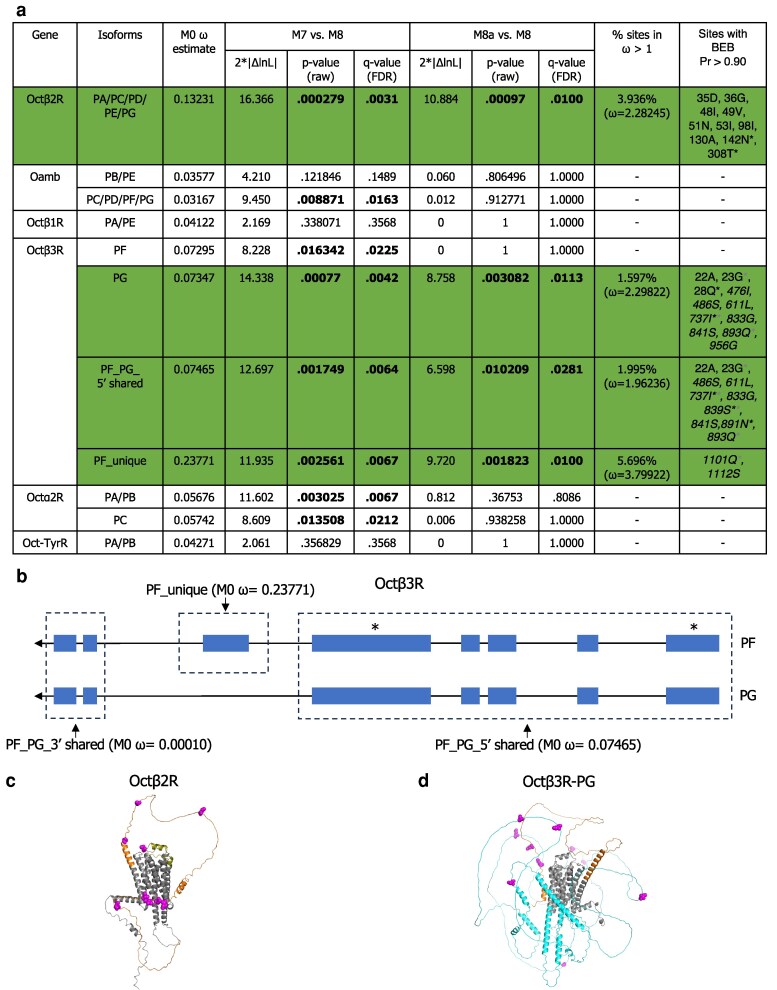
Octβ2R and Octβ3R are under recurrent positive selection in the *melanogaster* group. a) PAML test results for all 6 OA receptors across the *melanogaster* group. Significant *P*-values (raw *P*-values and Benjamini–Hochberg FDR-adjusted *P*-values [*q*-values, rounded to 4 decimal places]) are shown in bold, and coding sequences with significant evidence of positive selection are highlighted in green. The italicized sites in the last column (mapped to the *D. melanogaster* amino acid sequence) are located in the intracellular domain, while the others are extracellular. Asterisks in the last column indicate sites also detected as positively selected by MEME (for Octβ3R, gray asterisks indicate sites also detected in the PF isoform MEME analysis, black asterisks indicate sites detected in the PG isoform analysis, and sites marked with both gray and black asterisks were found in both MEME analyses) (Supplementary Files 4 and 5). BEB: Bayes empirical Bayes. b) Schematic of the PF and PG isoforms of Octβ3R. Boxes represent exons, lines indicate introns, and asterisks mark exons containing PSSs. The “PF_PG_5′ shared” and “PF_unique” regions tested in panel a), as well as the “PF_PG_3′ shared” exons, are outlined with dotted borders. M0 *ω* estimations for each region are indicated. c) Positively selected amino acid sites in Octβ2R (BEB posterior probability of >0.90) are shown in magenta and mapped onto the *D. melanogaster* protein sequence and AlphaFold-predicted structure. Nine sites are in the N-terminus (orange), and 1 site is in ECL2 (deep olive); both regions are extracellular. d) Positively selected amino acid sites in the PG isoform of Octβ3R (BEB posterior probability of >0.90) are shown in magenta and mapped onto the *D. melanogaster* protein sequence and AlphaFold-predicted structure. Three sites are in the N-terminus (orange; extracellular), and 8 sites are in ICL3 (cyan; intracellular).

Overall, OA receptors exhibit relatively low M0 *ω* estimates (*d*_N_*/d*_S_ across the entire gene), which may reflect their conserved roles in neuromodulatory and physiological processes. Octβ2R and Octβ3R have the 2 highest M0 *ω* estimates among them, and interestingly, we found significant evidence of positive selection acting on several amino acid sites within each of these 2 receptors (Fig. 3a). These genes have multiple isoforms identified, and with differences in CDS among some of these isoforms, which could lead to isoforms having differing results from site selection tests. We performed our analysis on all isoforms for our genes of interest that are sufficiently annotated across species. The results for different isoforms were largely consistent with one another; however, there was a difference in results for Octβ3R isoforms.

Under our criteria for the detection of positive selection, we identified sites under positive selection in the Octβ3R-PG isoform but not the PF isoform (Fig. 3a). The only difference in CDS between these isoforms is the presence of an extra exon in the PF isoform (Fig. 3b). Notably, the PSSs identified in the PG isoform using the BEB posterior probabilities from codeml's M8 output are all located in regions shared by both isoforms (Fig. 3a and b). To confirm the reliability of PSS detection, we performed an additional analysis focusing solely on this shared region (PF_PG_5′ shared), which yielded results consistent with those of the PG isoform, showing significant differences in both the M7/M8 and M8a/M8 comparisons, with most PSSs overlapping with those of PG (Fig. 3a). Analysis of the PF-unique exon also detected positive selection under both M7/M8 and M8a/M8 comparisons and exhibited the highest M0 *ω* estimate relative to the shared regions (Fig. 3a and b). Interestingly, when this exon is included in the full PF isoform, the signal of positive selection is lost. This apparent loss of significance may result from the unstructured nature of intracellular loop 3 (ICL3) encoded by the PF-unique exon, which is likely evolving under reduced constraint. Such variation could alter the underlying *ω* distribution and reduce the sensitivity of PSS detection in the full isoform. Moreover, the *P*-values for Octβ2R and Octβ3R-PG remain significant after Benjamini–Hochberg false discovery rate (FDR) correction (*q*-value; Fig. 3a), supporting the robustness of our results.

The PSSs in Octβ2R and Octβ3R are predominantly clustered within unstructured loops (Fig. 3c and d). In Octβ2R, the PSSs are primarily located in the N-terminus, with 1 additional site in extracellular loop 2 (ECL2) (Fig. 3c). Notably, in the human β2-adrenergic receptor, a predicted ortholog of Octβ2R, the N-terminus and ECL2 have been reported to be important for ligand binding and accessibility (Isin et al. 2012; Shahane et al. 2014). This suggests that Octβ2R may have evolved to optimize interactions with its ligand. In some *melanogaster* group species, Octβ2R may have evolved to bind OA with altered affinity. Alternatively, since OA is identical across species, this raises the intriguing possibility that Octβ2R could have adapted to bind an additional ligand. In Octβ3R, the PSSs are located in the N-terminus and predominantly in ICL3 (Fig. 3d). In class A GPCRs, ICL3 plays a crucial role in signal transduction and receptor activation, with its conformation and length influencing G protein accessibility and selectivity (Sadler et al. 2023). The adaptive evolution of ICL3 in Octβ3R may partially explain why Octβ3R is unable to fully compensate for Octβ2R's function in ovulation, unlike Octβ1R (Lim et al. 2014). Additionally, Octβ3R may have evolved to interact with different G proteins and/or acquired novel or specialized signaling functions in certain species.

To validate the PAML-based results, we performed complementary analyses, including assessment of mutational bias and additional model-based tests (MEME). To exclude mutational bias as a driver of elevated *ω* values, we examined codon usage bias, measured by the ENC, and GC content, including GC3, in the *melanogaster* group. Both genes show moderate codon usage bias (ENC: Octβ2R ∼42 to 50; Octβ3R-PG ∼41 to 49) and relatively stable GC content across species (Octβ2R: GC ∼0.55 to 0.58, GC3 ∼0.72 to 0.81; Octβ3R-PG: GC ∼0.58 to 0.61, GC3 ∼0.72 to 0.79) (Supplementary File 2), indicating that mutational bias is unlikely to explain the observed patterns. Finally, we used MEME analysis (Murrell et al. 2012) to investigate whether an alternate approach also detected PSSs in Octβ2R and Octβ3R. Unlike PAML, which tests for pervasive selection, MEME detects episodic selection using a different statistical framework. MEME identified more PSSs than PAML, and although only a few sites overlapped between the 2 analyses (Fig. 3a), the PSSs detected by either method were localized in similar regions of the proteins. Detailed discussion of these analyses and supporting data can be found in the supplement (Supplementary Files 3 to 5).

In addition, Octα2R and certain isoforms of Oamb also showed sites under positive selection in the M7/M8 comparison but lost significance in the M8a/M8 test (Fig. 3a). Since the M8a null model is an adaptation of M7 that includes an additional category of sites where *ω* = 1 (neutrally evolving), this result suggests that these sites are more likely evolving under neutral rather than adaptive selection. The possibility of redundancy with some of the other OA receptors could also contribute to relaxed selective constraint on Octβ2R and Octβ3R, allowing them to evolve more rapidly.

From a broader perspective, the observation that only some OA receptors have sites that have evolved under positive selection in distinct functional domains highlights the heterogeneous selective pressures shaping the evolution of this receptor family.

### Octβ2R does not contain PSSs in the *D. virilis*-*repleta* radiation nor in mosquitoes

**Fig. 4.**
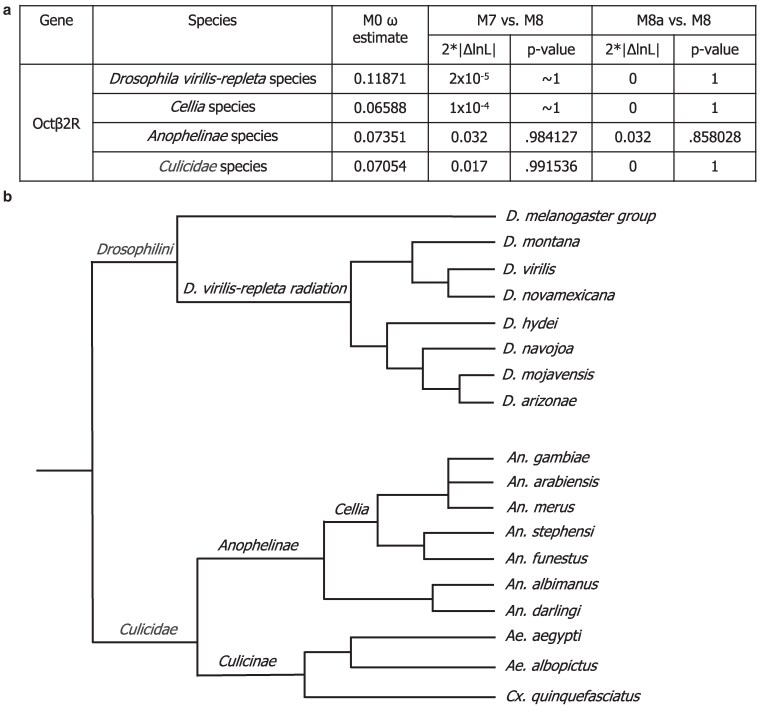
Octβ2R is highly conserved in the *D. virilis*-*repleta* radiation and in mosquitoes. a) PAML test results for Octβ2R across the *virilis*-*repleta* radiation and mosquito (*Cellia*, *Anophelinae*, and *Culicidae*) species. No significant differences were detected in any M7 vs. M8 or M8a vs. M8 comparisons, indicating that Octβ2R is under purifying selection in these groups. b) Phylogeny of the *virilis*-*repleta* radiation and mosquito species used in the PAML analyses, based on the species tree reported by Suvorov et al. (2022) and Neafsey et al. (2015). The *melanogaster* group is also included to illustrate relationships among the 3 clades in which Octβ2R was analyzed.

We were curious whether positive selection of Octβ2R was restricted to the *melanogaster* clade, or part of a broader pattern of its evolution. To explore this, we examined whether Octβ2R is also under positive selection in other clades. We first analyzed species from the *virilis*-*repleta* radiation, which last shared a common ancestor with the *melanogaster* group approximately 40 to 60 million years ago within the *Drosophila* genus (Russo et al. 1995; Tamura et al. 2004). Using PAML, we found that both the M7/M8 and more sensitive M8a/M8 comparisons showed no significant difference, suggesting that Octβ2R is under purifying selection in this clade (Fig. 4a). To further broaden our analysis, we extended our tests to mosquitoes, a more distantly related group of Dipterans. To minimize the risk of spurious signals of positive selection driven by synonymous site saturation due to excessive evolutionary distance, we adopted a stepwise approach to the PAML analyses. We began with the closely related *Cellia* species, where the M7/M8 and M8a/M8 comparisons revealed no evidence of positive selection (Fig. 4a and b). We then expanded our analysis to *Anophelinae* and subsequently to the broader *Culicidae* species (Fig. 4b), despite the divergence between *Culicinae* and *Anophelinae* exceeding 100 million years (Krzywinski et al. 2006). Across all comparisons, we consistently found no evidence of positive selection (Fig. 4a).

PSSs were detected in Octβ2R within the *D. melanogaster* group but not in the *D. virilis*-*repleta* radiation or mosquito species, suggesting a lineage-specific change in evolutionary selective pressure. One possibility could be that there is lineage-specific functionality in a process such as reproduction. Previous studies have shown that Octβ2R is essential for ovulation (Lim et al. 2014; Li et al. 2015) and is expressed in both the oviduct epithelium and OA neurons that project into the reproductive tract (Koon et al. 2011; Deshpande et al. 2022). *D. virilis* and *D. melanogaster* vary greatly in the coterie of seminal fluid proteins identified in their genomes and ejaculates (Garlovsky and Ahmed-Braimah 2023); several of these proteins have been observed to undergo rapid sequence divergence, sometimes driven by positive selection (Swanson et al. 2001a; Haerty et al. 2007; Wong and Wolfner 2012; Patlar et al. 2021). Among other hypotheses, it is intriguing to wonder whether rapid evolution of the female reproductive tract-expressed Octβ2R in the *melanogaster* clade might be driven by interaction with a rapidly evolving ejaculate protein in this clade, such as ovulin, which stimulates ovulation through modulating OA signaling (Aguadé et al. 1992; Tsaur and Wu 1997; Aguadé 1998; Tsaur et al. 1998; Heifetz et al. 2000; Wong et al. 2006; Rubinstein and Wolfner 2013) and is absent from species like *D. virilis* and *D. mojavensis*. However, experimental validation would be needed to confirm any potential interaction between *D. melanogaster* Octβ2R and ovulin, or any other protein.

### Conclusion

OA receptors are key modulators of physiology and behavior in insects, yet their evolutionary features remain largely unexplored. While their essential roles imply strong functional constraints, their involvement in reproduction-related processes may also subject them to shifting evolutionary selective pressures. In this study, we examined the molecular evolution of OA receptors across *Drosophila* species and uncovered signatures of both conservation and diversification. Most OA receptors were found as single-copy genes, consistent with stringent functional constraints acting on them. However, we identified copy number changes in 2 species, *D. sechellia* (Octα2R) and *D. busckii* (Octβ3R), which may reflect lineage-specific adaptations. Among the 6 OA receptors, Octβ2R and Octβ3R within the *D. melanogaster* clade evolve under positive selection and contain PSSs in functionally distinct regions of the GPCR domain. This heterogeneity suggests that different OA receptors are subject to distinct selective pressures across the genus *Drosophila* and between paralogs, likely reflecting divergent roles, molecular partners, or regulatory mechanisms. Notably, the detection of positive selection in the ligand binding-involved regions of Octβ2R within the *melanogaster* group, but not in other lineages, raises the possibility of lineage-specific selective pressure on ligand interaction. Together, our findings illustrate the diverse evolutionary trajectories of a closely related receptor family and motivate future functional studies aimed at understanding the molecular and ecological roles of OA receptors in *Drosophila*.

## Supplementary Material

jkaf289_Supplementary_Data

## Data Availability

PAML analysis data, MEME output files and jsons, and PSE files (PyMOL session files) showing the structures of Octβ2R and Octβ3R with annotated PSSs and functional domains (extracellular, transmembrane, and intracellular regions) are provided in the supplementary files. Supplemental material available at G3 online.
