# Supplementary material for: Molecular evolution of octopamine receptors in Drosophila
Source: G3 (Bethesda). 2025 Dec 6;16(2):jkaf289. doi: 10.1093/g3journal/jkaf289 (PMC12869069; doi:10.1093/g3journal/jkaf289)
Supplement: jkaf289_Supplementary_Data [file jkaf289_supplementary_data.zip › Figure_S1_G3-2025-406309.pdf]

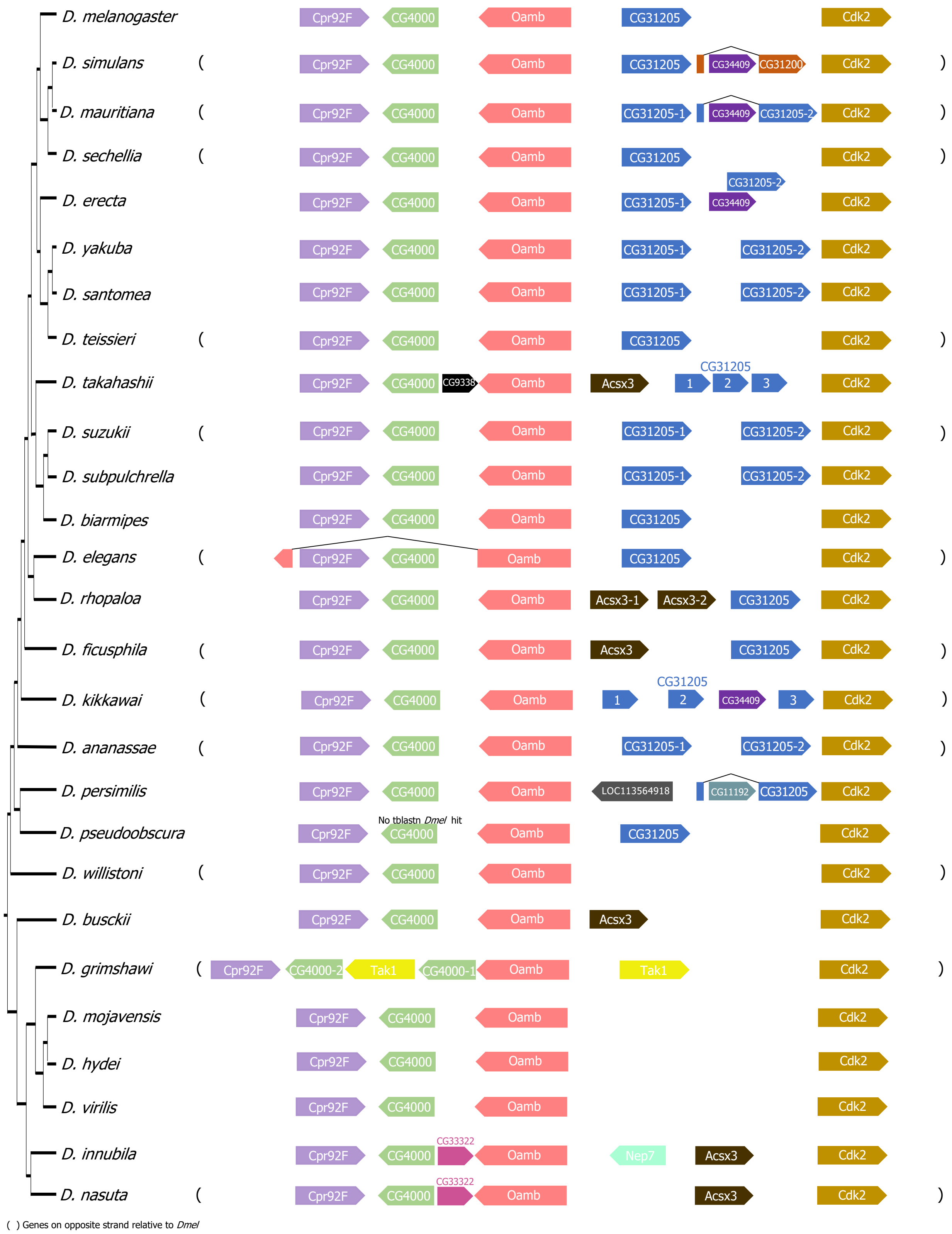

Figure S1. Syntenic region of *Oamb* across *Drosophila* species. The phylogeny is based on Suvorov et al. (2022) and Hopkins et al. (2024). Surrounding gene names correspond to orthologs in *D. melanogaster*.
