# Supplementary material for: Molecular evolution of octopamine receptors in Drosophila
Source: G3 (Bethesda). 2025 Dec 6;16(2):jkaf289. doi: 10.1093/g3journal/jkaf289 (PMC12869069; doi:10.1093/g3journal/jkaf289)
Supplement: jkaf289_Supplementary_Data [file jkaf289_supplementary_data.zip › Figure_S4_G3-2025-406309.pdf]

+: detected -: not detected

	Octβ2R	Oamb		Octβ1R			Octβ3R				Octα2R		Oct-TyrR
	PA/PC/PD/PE/PG	PB/PE	PC/PD/PF/PG	PA/PE	PB	PC	PF	PG	PJ	PK	PA/PB	PC	PA/PB
D. ficusphila	+	+	+	+	-	-	+	+	-	-	+	-	+
D. elegans	+	+	+	+	-	-	+	+	-	-	+	+	+
D. rhopaloa	+	-	+	+	-	-	+	+	-	-	+	+	+
D. takahashii	+	+	+	+	+	-	+	+	-	-	+	+	+
D. biarmipes	+	+	+	+	-	-	+	+	-	-	+	+	+
D. suzukii	+	+	+	+	-	-	+	+	-	-	+	+	+
D. subpulchrella	+	+	+	+	+	+	+	+	-	-	+	+	+
D. melanogaster	+	+	+	+	+	+	+	+	+	+	+	+	+
D. simulans	+	+	+	+	+	-	+	+	-	-	+	+	+
D. mauritiana	+	+	+	+	+	-	+	+	-	-	+	+	+
D. sechellia	+	+	+	+	-	-	+	+	-	-	Duplication		+
D. santomea	+	+	+	+	+	-	+	+	-	-	+	+	+
D. yakuba	+	+	+	+	+	+	+	+	-	-	+	+	+
D. teissieri	+	+	+	+	-	-	+	+	+	+	+	-	+
D. erecta	+	+	+	+	-	-	+	+	-	-	+	+	+

Figure S4. Species within the *melanogaster* group used for PAML analyses of OA receptors are shown in gray (+: Orthologs detected by Gnomon prediction. -: Orthologs not detected by Gnomon prediction.). For receptors with multiple different isoforms, only those detected in more than 10 species were included in the analysis. *D. sechellia* was excluded from Octα2R analysis due to the tandem duplication event in this species.
