# Supplementary material for: Molecular evolution of octopamine receptors in Drosophila
Source: G3 (Bethesda). 2025 Dec 6;16(2):jkaf289. doi: 10.1093/g3journal/jkaf289 (PMC12869069; doi:10.1093/g3journal/jkaf289)
Supplement: jkaf289_Supplementary_Data [file jkaf289_supplementary_data.zip › Supplementary Files/Supplementary File 3.docx]

**MEME and Codeml PSSs Analyses are Consistent**

We used both MEME to determine if an alternative method to codeml would detect PSSs in Octβ2R and Octβ3R. MEME detected PSSs in both PF and PG Isoforms of Octβ3R, and in Octβ2R. MEME detected far more sites than what were detected by codeml. Only a few detected PSSs in codeml overlapped with those detected by MEME. However, PSSs from both methods were identified within similar regions of the proteins. For Octβ2R, the majority of PSSs were detected in the N terminus (14) and followed by extracellular loop 2 (3), the regions where all codeml PSSs were detected (codeml: 9 PSSs in N-term, 1 in ECL2). MEME also detected PSSs in ECL1 (1) and ECL3 (1) (File S4). For Octβ3R, we saw a similar result where similar regions were shown to contain PSSs despite many of the detected PSSs not being the same between methods. For both MEME and codeml analysis of Octβ3R (PG), all PSSs were detected in the N terminus (codeml:3, MEME:12) and intracellular loop 3 (codeml:8, MEME:13). The PF isoform of Octβ3R has a longer intracellular loop 3 due to having an additional exon, within this region MEME detected 6 PSSs when running against an alignment of the full PF isoform. The full-length PF isoform was predicted by MEME to have 34 PSSs (File S5). While it is unsurprising that two different methods would differ in what PSSs are detected, it is also reassuring that MEME analysis validates our results of detecting positive selection in Octβ2R and Octβ3R and what regions of these proteins contain PSSs. While codeml identifies PSSs under pervasive positive selection, MEME detects sites under episodic positive selection. Further, while our codeml analysis removes gapped regions of the alignment, analysis by MEME does not. Many of the PSSs identified by MEME are within regions that are poorly aligned and contain gaps, potentially making these predictions less reliable. MEME’s inclusion of poorly aligned gapped regions in the analysis and MEME detecting sites under episodic rather than pervasive selection are both likely major contributors to why MEME identified more sites under positive selection than our codeml analysis.

In the supplementary data, we have annotated PSSs from MEME and codeml on the protein alignments used in all analyses (File S4, S5). Further, we have included in the supplement the jsons and CSV tables output by MEME where positive selection was detected.
