# Supplementary material for: Molecular evolution of octopamine receptors in Drosophila
Source: G3 (Bethesda). 2025 Dec 6;16(2):jkaf289. doi: 10.1093/g3journal/jkaf289 (PMC12869069; doi:10.1093/g3journal/jkaf289)
Supplement: jkaf289_Supplementary_Data [file jkaf289_supplementary_data.zip › Supplementary Files/Supplementary File 6.docx]

**Supplementary methods for ortholog identification and synteny analysis**

As described in the Materials and Methods section (Synteny analysis and tandem duplication detection), we used tblastn searches to identify orthologs in species other than *D. melanogaster* and confirmed their identity with reciprocal best blast (tblastn) tests (RBB). Relevant data are provided in File S1.

In most cases, the orthologs correspond to the top tblastn hit (or hits in cases of duplication). However, there are exceptions. For example, in *D. busckii*, duplicated Octβ3R corresponds to the 4th and 5th hits, while the top hits represent Octβ1R or Octβ2R. However, we are still certain in this case that the identified locus is a paralog of Octβ3R due to being located in Octβ3R’s syntenic location and when these sequences are queried against the *D. melanogaster* transcriptome via tblastn, Octβ3R is the top hit. In other cases, the top hits may pass the RBB test but fall outside the syntenic region. Because our analyses focus on OA receptor syntenic regions, only hits within those regions are included in File S1, and their ranks from the initial tblastn query are noted. This approach inevitably excludes some orthologs that have relocated due to evolutionary events such as gene duplications and rearrangements. For example, the Hsp70 family, neighboring genes of Octβ2R and Octβ3R, often show orthologs outside the Octβ2R/Octβ3R syntenic region. Since our aim was to assess tandem duplication events of OA receptors within their syntenic context, such relocated copies were not considered.

Similarly, in cases where both the first and second hits passed the RBB test but only the first fell within the syntenic region, we reported only the syntenic hit and did not provide further notes. When a miscRNA appeared as the top hit, it was ignored in favor of the next protein-coding hit. By default, entries in File S1 correspond to the top protein-coding hits from the initial tblastn query; exceptions are annotated in the relevant lines of File S1.
